# TM4SF1 promotes EMT and cancer stemness via the Wnt/β-catenin/SOX2 pathway in colorectal cancer

**DOI:** 10.1186/s13046-020-01690-z

**Published:** 2020-11-05

**Authors:** Qiang Tang, Jinhuang Chen, Ziyang Di, Wenzheng Yuan, Zili Zhou, Zhengyi Liu, Shengbo Han, Yanwei Liu, Guoguang Ying, Xiaogang Shu, Maojun Di

**Affiliations:** 1 Tianjin Medical University Cancer Institute and Hospital, National Clinical Research Center for Cancer, Key Laboratory of Cancer Prevention and Therapy, Tianjins Clinical Research Center for Cancer, Tianjin, 300060 China; 2 Department of General Surgery, Taihe Hospital, Hubei University of Medicine, Shiyan, Hubei Province China; 3 Department of Gastrointestinal Surgery, Union Hospital, Tongji Medical College, Huazhong University of Science and Technology, Wuhan, China

**Keywords:** TM4SF1, Colorectal cancer, SOX2, Stemness, EMT, Wnt/β-catenin

## Abstract

**Background:**

Transmembrane 4 L six family member 1 (TM4SF1) is upregulated in several epithelial cancers and is closely associated with poor prognosis. However, the role of TM4SF1 and its potential mechanism in colorectal cancer (CRC) remain elusive.

**Methods:**

We investigated the expression of TM4SF1 in the Oncomine, the Cancer Genome Atlas (TCGA) and Gene Expression Omnibus (GEO) databases and confirmed the results by immunohistochemistry (IHC), qPCR and Western blotting (WB) of CRC tissues. The effect of TM4SF1 on the epithelial-to-mesenchymal transition (EMT) and cancer stemness of CRC cells was investigated by Transwell, wound healing and sphere formation assays. A series of in vitro and in vivo experiments were conducted to reveal the mechanisms by which TM4SF1 modulates EMT and cancer stemness in CRC.

**Results:**

TM4SF1 expression was markedly higher in CRC tissues than in non-tumour tissues and was positively correlated with poor prognosis. Downregulation of TM4SF1 inhibited the migration, invasion and tumour sphere formation of SW480 and LoVo cells. Conversely, TM4SF1 overexpression significantly enhanced the migration, invasion and tumoursphere formation potential of CRC cells, Additionally, TM4SF1 silencing inhibited the EMT mediated by transforming growth factor-β1 (TGF-β1). Mechanistically, gene set enrichment analysis (GSEA) predicted that the Wnt signalling pathway was one of the most impaired pathways in TM4SF1-deficient CRC cells compared to controls. The results were further validated by WB, which revealed that TM4SF1 modulated SOX2 expression in a Wnt/β-catenin activation-dependent manner. Furthermore, we found that knockdown of TM4SF1 suppressed the expression of c-Myc, leading to decreased c-Myc binding to the SOX2 gene promoter. Finally, depletion of TM4SF1 inhibited metastasis and tumour growth in a xenograft mouse model.

**Conclusion:**

Our study substantiates a novel mechanism by which TM4SF1 maintains cancer cell stemness and EMT via the Wnt/β-catenin/c-Myc/SOX2 axis during the recurrence and metastasis of CRC.

## Background

Colorectal cancer (CRC) is one of the most prevalent malignancies worldwide and remains the third leading cause of global cancer-related morbidity and mortality [1]. Distant invasion and metastasis are responsible for up to 90% of CRC-associated mortalities. Although great improvements in clinical diagnosis and comprehensive therapy have partly prolonged survival, the prognosis of CRC patients with metastasis remains poor [2].

Transmembrane 4 L6 family member 1 (TM4SF1) is the founding member of the tetraspanin-related L6 family (TM4SF) characterized by four highly conserved transmembrane domains, two extracellular loops and a small intracellular loop [3, 4]. TM4SF1 was initially defined as a tumour-associated antigen that shares certain tetraspanin functions, including stabilization of cell signalling complexes and roles in cell proliferation, adhesion, and motility [5–7]. Immunostaining of nanopodia with both a light microscope and an electron microscope showed TM4SF1 expression in a regularly spaced, banded pattern, forming TM4SF1-enriched domains (TMEDs) that anchor nanopodia to regulate cell movement [4, 8]. Studies have confirmed that TM4SF1 is highly expressed in various epithelial cancer cells, including pancreatic, liver, lung and bladder cancers [9–11]. A previous study reported that TM4SF1 was also upregulated in endothelial cells lining angiogenic tumour blood vessels, and they also found that TM4SF1 serves as a molecular organizer that is essential for the formation of nanopodia and the maturation of angiogenesis [12]. Chi-Iou Lin and colleagues reported that TM4SF1 monoclonal antibodies that reacted with the extracellular loop-2 (EL2) of TM4SF1 could effectively eliminate the human vascular network in Matrigel implants. Recently, most studies about TM4SF1 have mainly focused on TM4SF1 functions as a direct target of some miRNAs (miR-141, miR-9 miR-206) and its biological function in cancer cells [13–15]. However, the molecular mechanisms of TM4SF1 action in CRC remain elusive. Therefore, further investigation is warranted to identify the downstream target genes of TM4SF1 that are involved in CRC development.

Epithelial-to-mesenchymal transition (EMT) plays a critical role in tumourigenesis and metastasis [16]. During EMT, epithelial tumour cells undergo distinct morphological and phenotypical changes, including loss of tight junctions, cell polarity and cytoskeletal reorganization, which renders cells more invasive properties and phenotypes [17, 18]. Cancer stem cells (CSCs) are capable of self-renewal, asymmetric cell division, resistance to apoptosis, tumourigenicity and high metastatic potential [19, 20]. Abundant evidence indicates that CSCs are involved in tumour invasion, metastasis, and radiotherapy or chemotherapy-induced resistance [21, 22]. In addition, accumulating studies have revealed that EMT plays an important role in the enrichment of cells with CSC properties, which is believed to be the origin of cancer progression. Therefore, all these findings may represent a novel approach for research in CRC. In this study, we found that TM4SF1 was significantly upregulated in CRC and positively correlated with poor prognosis. In addition, TM4SF1 silencing suppressed CRC stemness and EMT, which are critical for CRC invasion and metastasis. Mechanistically, we found that TM4SF1 promotes cell metastasis and maintains the phenotypes of EMT and cancer stemness via the Wnt/β-catenin/c-Myc/SOX2 pathway in CRC.

## Materials and methods

### Specimens and immunohistochemistry (IHC)

The cancer tissues (T) and paired adjacent tissues (N) were obtained from the Gastrointestinal Surgery Department of Union Hospital, Tongji Medical College, Huazhong University of Science and Technology, Wuhan, China, from July 2012 to April 2017. None of the patients received any radiochemotherapy before the operation. Immunohistochemistry staining was performed as described elsewhere [23], and specific antibodies were used as follows: TM4SF1, β-catenin (1:100 dilution, Abcam, USA) and SOX2, CD133 (1:80 dilution; Abcam, USA). The expression of TM4SF1 was evaluated according to the intensity of the staining (0, 1+, 2+ and 3+) and the percentage of positive cells, which was scored as 0 (0%), 1 (1–25%), 2 (26–50%), 3 (51–75%) or 4 (76–100%). The staining index (SI) was calculated as follows: SI = (intensity score in 1) × (positive staining score in 2). SI < 3 was classified as low expression, while SI ≥4 was classified as high expression. Furthermore, the study was approved by the Human Subjects Protection Committee of the Tongji Medical College, Huazhong University of Science and Technology, China.

### Cell culture and reagents

The CRC cell lines LoVo and NCM460 were cultured in DMEM (Boster, China). The HCT116, SW480, RKO, FHC, and DLD1 cell lines were cultured in RPMI 1640 medium (HyClone, USA). All of the cell lines were cultured in medium supplemented with 10% foetal bovine serum (FBS, ScienceCell). Primary antibodies against the proteins TM4SF1, N-cadherin, vimentin, SOX2, MMP9, CD133, Par3 and β-catenin were purchased from Abcam (USA), while those against E-cadherin, c-Myc, and ZO1 were purchased from Santa Cruz (USA), and those against Smad2 and CD44 were purchased from Sigma (USA). The second antibody, affinity-purified, biotinylated rabbit anti-rat IgG, was purchased from Sigma (USA). Accession numbers are available in Table S1.

### Lentiviral vector, plasmids, shRNA, and transfection conditions

To transiently silence the expression of TM4SF1, siRNAs (Ribo-Bio, Guangzhou, China) were transfected into SW480 and LoVo cells by using Lipofectamine 3000. TM4SF1 shRNA (Shanghai Gene Chem Co, Ltd., China) was used to establish stable cell lines to knock down the expression of TM4SF1. The SOX2, TM4SF1 cDNA, c-Myc cDNA, and β-catenin cDNA plasmids (Shanghai GeneChem Co, Ltd., China) were used to upregulate the expression of SOX2, c-Myc and β-catenin. All transfection procedures were performed according to the manufacturer’s instructions.

### qPCR and Western blotting (WB)

Real-time PCR was performed as described elsewhere [9]. The primers for RT-PCR were designed in our laboratory and synthesized by Sangon (Shanghai, China, Table S2). mRNA expression was quantitated using the 2-(△Ct sample - △Ct control) method. WB and band density analyses were conducted as described previously [23]. Whole cells were lysed in RIPA buffer containing phosphatase inhibitor and protease inhibitor cocktail. The bicinchoninic acid (BCA) protein assay kit (Pierce, Rockford, USA) was used to determine protein concentrations. Antibodies against proteins are listed in Supplementary Table S1. The bands of interest in the Western blots were normalized to GAPDH or tubulin.

### Transwell assays

Migration and invasion were examined by Boyden chamber assay (8-μm pore). CRC cells were resuspended in 200 μL FBS-free medium (1 × 10^5^ cells) and added to the top chamber (BD, USA). Medium supplemented with 10% FBS was added to the lower chamber. After 24 h, the cells were fixed and stained, and the number of cells in six randomly selected fields was counted under the microscope. The invasion assay was similarly conducted with a modified Boyden chamber whose upper chamber was coated with Matrigel (BD Bioscience, USA), and the rest of the protocol was performed in a similar manner as the migration assay.

### Wound healing and cell counting Kit-8 (CCK-8) viability assays

Cells (1 × 10^5^) were cultured in 6-well plates. After 16 h, the complete medium was replaced with a low concentration of serum fresh medium (2%). Consistently shaped wounds were scratched with a 10-μL pipette tip across each well after the cells reached 90% confluence, based on a technique described elsewhere [24]. The cells were gently washed with PBS twice to remove loose cells, and serum-free medium was added. To ensure that the wounds with the same wound area were comparable, multiple positioning marks were made at the center of the denuded surface. The scratch zones were photographed by inverted microscopy at 0 and 24 h. Axio Vision Rel. 4.8 software was used for the measurements and to determine the migrating ability of cancer cells. The data presented were repeated three times.

Cell viability was examined using a CCK-8 assay (Sevenbio, China). Cells were seeded in 96-well plates at a density of 4 × 10^3^ cells per well in 200 μL medium for 24 h, 48 h and 72 h. The absorbance was detected at 450 nm after the cells were treated with 10% CCK-8 at 37 °C for 2 h. Cell viability was calculated as a ratio of optical density (OD) values of drug-treated samples to those of controls.

### Tumour sphere formation

Tumour sphere formation was performed as described elsewhere [25, 26]. Cells (4 × 10^4^) were seeded in six-well ultra-low attachment plates per well (Corning, NY, USA) in sphere formation medium: serum-free DMEM/F-12 (Invitrogen) supplemented with β-FGF (10 ng/mL, Invitrogen), B27 (20 ng/mL, Invitrogen), human EGF (20 ng/mL, Invitrogen) and IGF (20 ng/ml, Cell Signaling). Cells were subsequently cultured at 37 °C in an atmosphere containing 5% CO2 to form tumourspheres. After 10–14 days, the images of cells were captured by inverted microscopy at a magnification of × 100 or × 200. The number of tumourspheres was counted and plotted, and the percentage of tumourspheres with diameters of 50–100 μm, 100–150 μm or > 150 μm was calculated and plotted using ImageJ software.

### Immunoflorescence (IF) staining

The IF assay was carried out as described previously [27]. Cells were cultured on coverslips in 24-well plates for 24 h, fixed with 4% formaldehyde, blocked with 5% bovine serum albumin, and permeabilized with 0.5% Triton X-100. After that, the washed cells were incubated with polyclonal antibodies against TM4SF1 (Abcam, 1:50), SOX2 (Abcam, 1:80), Zo1 (Abcam, 1:100), and vimentin (Abcam, 1:50), followed by incubation with mouse/goat anti-rabbit antibodies labelled with Cy3 (Beyotime Institute of Biotechnology, 1:800). After incubation with DAPI (Biosharp Biotech, Hefei, China, 1:1000) for 5 min, the cells were observed under a fluorescence microscope within 4 h.

### Chromatin immunoprecipitation (ChIP) assays

We performed ChIP assays following the instructions of the EZ-ChIP™ kit (Millipore, Billerica, MA). The following antibodies were used for immunoprecipitation: rabbit anti-c-Myc (Abcam, USA) and rabbit anti-IgG (Abcam, USA). The ChIP DNA sample or 1% total input (5 μL) was precipitated, washed, dried, and resuspended in water. The enrichment of the specific amplified region was analysed by qRT-PCR.

### Tumour xenograft and metastasis in vivo

Male 4-week-old nude mice were purchased from Beijing HFK Bioscience Co., Ltd. (Beijing, China). Stably transfected cells were injected into the flank of the mice (20 mice in each group). We measured the size of xenografts using callipers (calculated volume = shortest diameter^2^ *longest diameter/2) weekly. The subcutaneous tumours were harvested at 4–5 weeks and then measured and weighed. Then, tumour tissue sections were stained by immunohistochemistry. The metastasis models were induced by tail vein injection as described in previous studies [23]. The mice were anesthetized by chloral hydrate (4%, 0.2 ml/20 g) and sacrificed by cervical dislocation and then their lung tissues were collected. The number and size of lung metastatic tumors were scored and analyzed. The experiment was completed in the Surgery Experimental Animal Center of Tongji Medical College and was approved by the Institutional Animal Care and Use Committee of Tongji Medical College, Huazhong University of Science and Technology.

### Statistical analysis

All of the experiments were independently performed at least three times, and all results are presented as the mean ± standard deviation. Pearson’s correlation coefficient analysis, the Kaplan-Meier plot, and logistic regression analyses were performed to assess the covariates of TM4SF1 expression. GraphPad Prism (version 6; La Jolla, CA, USA) and SPSS statistics software (version 23; IBM, Armonk, NY, USA) were used for statistical analysis. *P ≤* 0.05 was deemed to be statistically significant.

## Results

### High TM4SF1 expression was significantly associated with poor prognosis

**Fig. 1:**
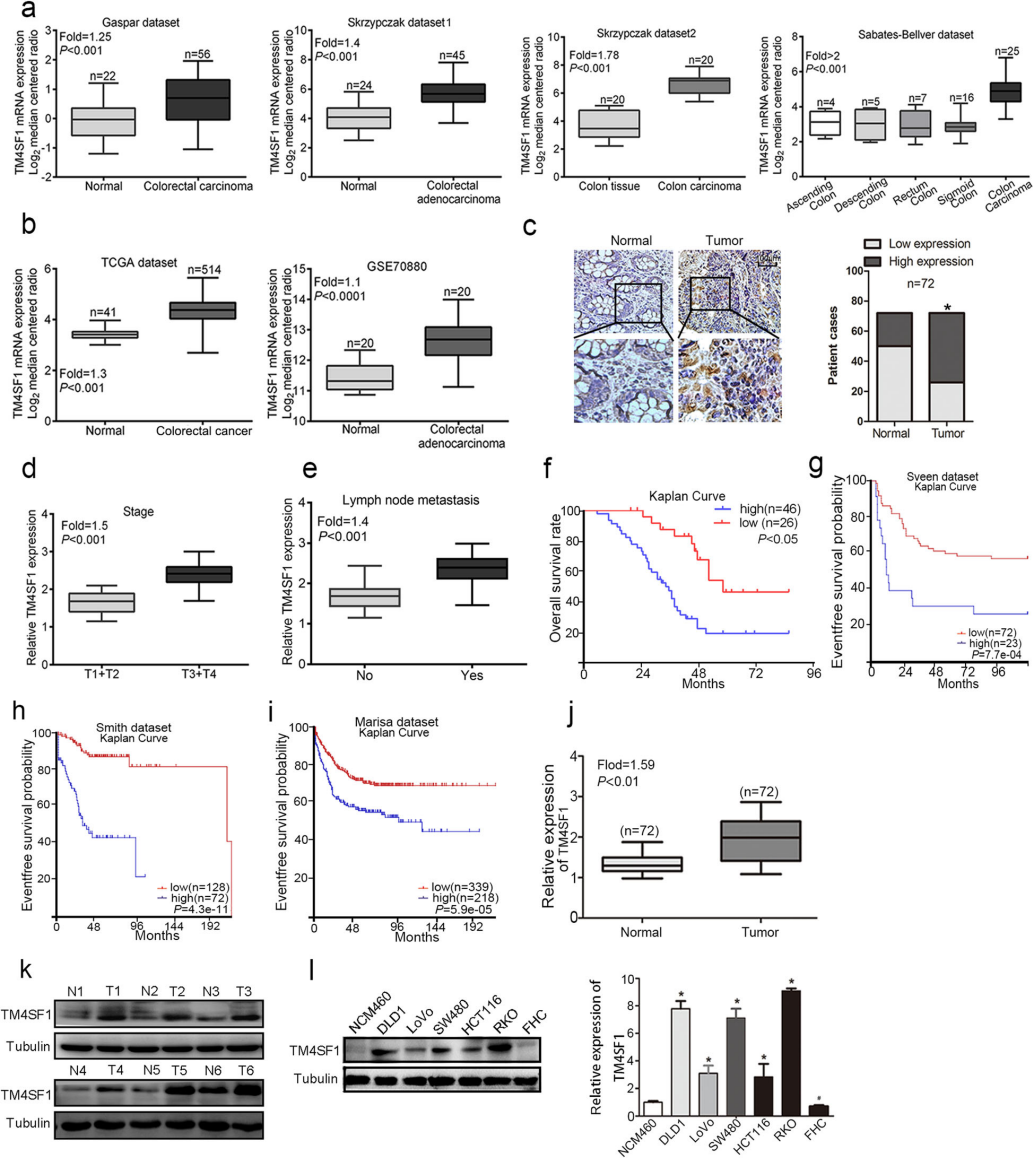
High TM4SF1 expression was significantly associated with poor prognosis. **a**, **b** Data from the Oncomine, TCGA and GEO databases showed that TM4SF1 was upregulated in CRC tissues compared to normal controls. **c** Immunohistochemical staining for TM4SF1 in CRC tissues and peritumoural normal tissues (*n* = 72). A total of 69% (50/72, SI ≥ 4) of CRC tissues were positive for TM4SF1 expression, while 31%(22/72, SI ≥3) of normal tissues were positive for TM4SF1. **d**, **e** High TM4SF1 expression showed a significant positive association with T stage and lymph node metastasis. **f** Kaplan-Meier survival analysis of TM4SF1 expression in the present study. **g**-**i** The overall survival curve was plotted by Kaplan-Meier Plotter in the R2 Genomics Analysis Platform (https://hgserver1.amc.nl/cgi-bin/r2/main.cgi; the study conducted by Sveen, Smith and Marisa). **j**-**k** qRT-PCR and WB analysis revealed that TM4SF1 was upregulated in CRC tissues (*P < 0.05). **l** WB and qPCR analysis of TM4SF1 expression in CRC cell lines (HCT116, SW480, DLD1, LoVo, RKO) and normal colon mucosal epithelial cells (NCM460 and FHC) *P < 0.05

We investigated the expression of TM4SF1 (cancer vs. normal) in the Oncomine (https://www.oncomine.org), The Cancer Genome Atlas (TCGA, https://tcgadata.nci.nih.gov/tcga/) and the Gene Expression Omnibus (GEO) (https://www.ncbi. nlm.nih.gov/gds) databases, and the results suggested that TM4SF1 was significantly upregulated in CRC compared with in adjacent normal tissues (*P* < 0.01, Fig. 1a, b). The IHC examination revealed an obvious abundance of TM4SF1 protein in CRC tissues (Fig. 1c). In addition, high TM4SF1 expression was significantly correlated with tumour T stage and lymph node metastasis (Fig. 1d, e, *P* < 0.01) but not with age, sex, tumour size, or tumour differentiation (*P* > 0.05, Table 1**)**. Moreover, Kaplan-Meier analysis indicated that CRC patients with elevated expression of TM4SF1 suffered from poor survival (Fig. 1f, *P* < 0.01), which was consistent with the studies conducted by Sveen, Smith and Marisa in the R2 Genomic Analysis Platform (Fig. 1g-i, *P* < 0.01, https://hgserver1.amc.nl/cgi-bin/r2/main.cgi). As shown in Fig. 1j, k TM4SF1 was significantly upregulated in CRC tissues compared with normal tissues (fold change = 1.59, *P* < 0.01). Then, we found that the expression of TM4SF1 in CRC cell lines (HCT116, LoVo, RKO, SW480, DLD1) was higher than that in normal colon epithelial cells (NCM460 cells and FHC cells, Fig. 1l).

**Table 1:** Clinicopathological characteristics of patients

Clinicopathologic features		No. of patients	TM4SF1 expression		***p***
			Positive	Negative	
Age (years)	≥60	40	25	15	0.784
	< 60	32	21	11	
Gender	Male	45	31	14	0.254
	Female	27	15	12	
Sizes	< 3	32	17	15	0.089
	> 3	40	29	11	
Pathologic T stage	T1 + T2	30	14	16	0.01*
	T3 + T4	42	32	10	
Vascular invasion	Yes	50	33	17	0.60
	No	22	13	9	
Lymph node metastasis	Yes	47	35	12	0.01*
	No	25	11	14	
Tumor differentiation	Poorly	20	16	4	0.37
	Moderate	36	24	12	
	Well	16	6	10	
Liver metastasis	Yes	31	24	7	0.038*
	No	41	22	19	

* Statistically significant

### TM4SF1 promotes cell migration, invasion and proliferation in CRC cells

**Fig. 2:**
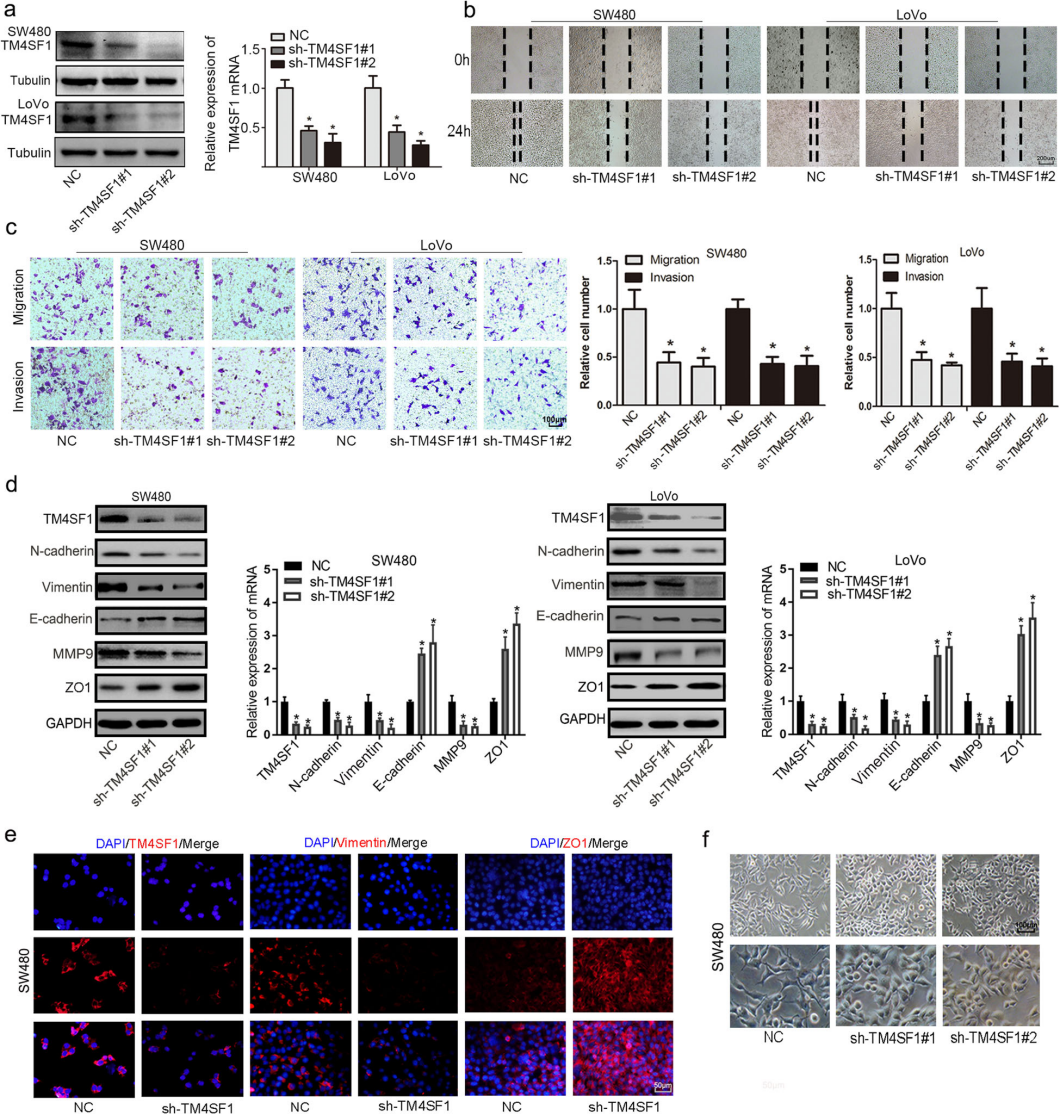
TM4SF1 deficiency suppresses the migration and invasion of CRC cells. **a** WB and qRT-PCR analysis of the efficiency of sh-TM4SF1 and sh-Control (NC) transfection in SW480 and LoVo cells. **b** Wound healing assays of cell migration in SW480 and LoVo cells. The images of wound closure are presented at the indicated number of hours after scratching (0, 24 h). **c** Transwell assays were performed to examine the potential migration and invasion of TM4SF1 KD cells or negative control cells. **d** WB and qRT-PCR analysis of the expression of EMT markers in CRC cells transfected with sh-TM4SF1. **e** Immunofluorescence staining showed the changes in the expression of EMT-associated genes vimentin and ZO1 (red) in SW480 cells. Nuclei were counterstained with DAPI (blue). **f** Morphological change of SW480 cells transfected with sh-TM4SF1 and NC. *P < 0.05

Specific shRNAs (sh-Control, sh-TM4SF1#1/2) and TM4SF1 plasmids were transfected into SW480 and LoVo cells, and the expression of TM4SF1 was confirmed by qPCR and WB (Fig. 2a and Fig. S1a). Then, a wound healing assay indicated that depletion of TM4SF1 significantly suppressed scratch wound healing and TM4SF1 overexpression enhanced the migration of CRC cells (Fig. 2b and Fig. S1b). Consistent with these results, the Transwell assay confirmed that TM4SF1 silencing inhibited the migration and invasion of SW480 and LoVo cells (Fig. 2c). In contrast, cells with TM4SF1 overexpression exhibited more aggressive migratory and invasive potential (Fig. S1c). qPCR and WB analysis showed that TM4SF1 knockdown increased the expression of E-cadherin and ZO1 and decreased the expression of vimentin, N-cadherin and MMP9 (Fig. 2d), while TM4SF1 overexpression increased the expression of vimentin, N-cadherin, and β-catenin and decreased the expression of E-cadherin (Fig. S1d). As shown in Fig. 2e, sh-TM4SF1-transfected SW480 cells presented an increased distribution of ZO-1 on the cell membrane and a decreased expression of vimentin in the cytoplasm or nucleus. Interestingly, TM4SF1-silenced SW480 cells showed a conversion from a spindle-like mesenchymal phenotype with obvious characteristics of the interstitial cells to a cobblestone-like shape, as observed under a phase contrast microscope **(**Fig. 2f**)**. In contrast, TM4SF1 overexpression resulted in decreased expression of ZO-1 and increased expression of vimentin (Fig. S1e). And TM4SF1-overexpressing cells exhibited a more mesenchymal phenotype than control cells, as observed under a phase contrast microscope (Fig. S1f).

### TM4SF1 is involved in the process of EMT induced by TGF-β1

**Fig. 3:**
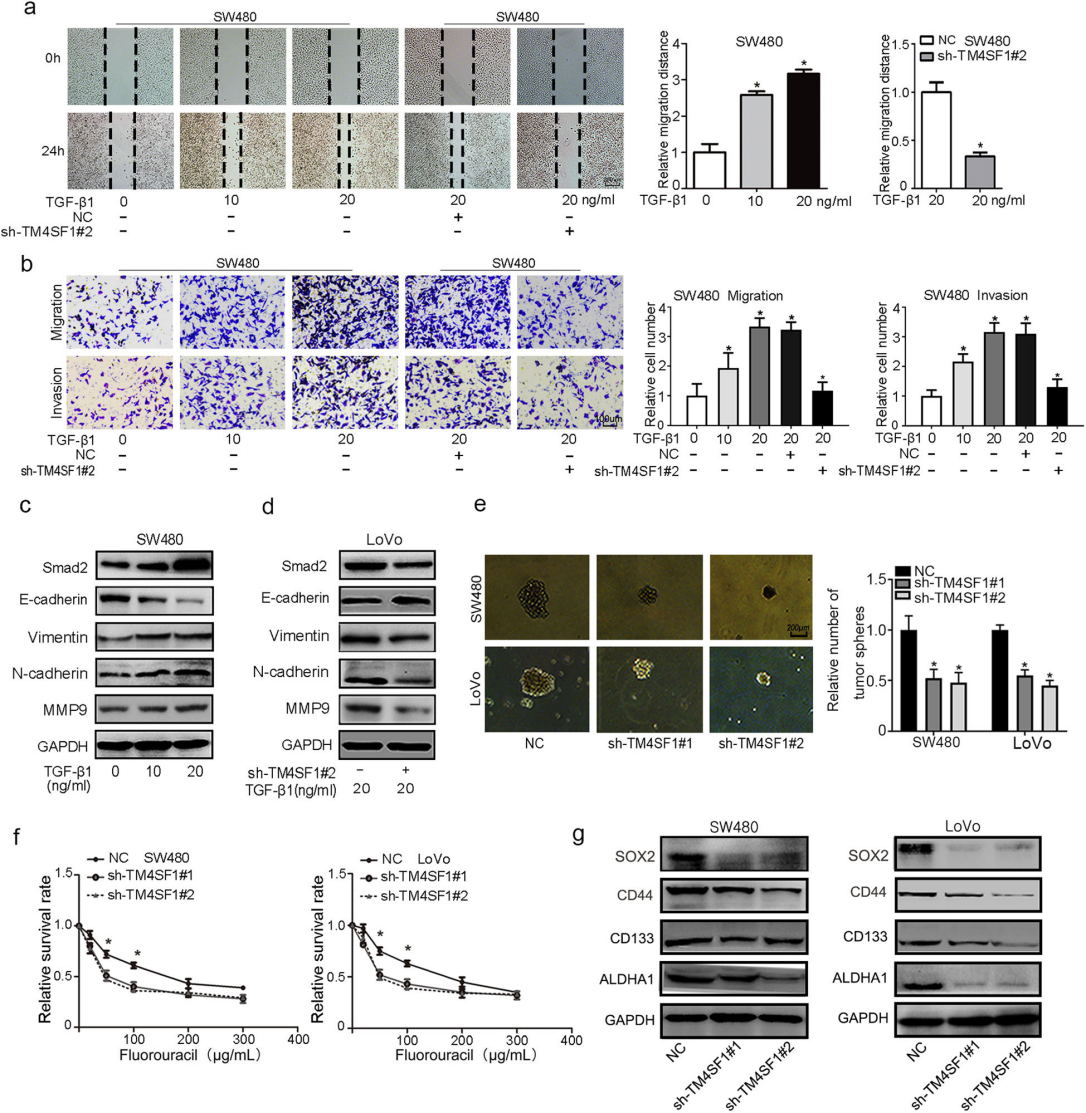
TM4SF1 is involved in TGF-β1-induced EMT, and modulates the stemness properties. **a** Wound healing assays showed that TGF-β1 enhanced the migration potential. TM4SF1 silencing decreased the migration of TGF-β1-treated SW480 cells. **b** Transwell assays showed that TGF-β1 enhanced the migration and invasion potential. TM4SF1 silencing suppressed the migration and invasion of TGF-β1-treated SW480 cells. **c** WB analysis showed that TGF-β1 increased the expression of Smad2, and EMT markers (vimentin, N-cadherin, MMP9). **d** TM4SF1 knockdown downregulated the expression of EMT markers in TGF-β1-treated LoVo cells. **e** Tumoursphere assay of CRC cells transfected with sh-TM4SF1 and NC. **f** CCK-8 assay showed that CRC cells with sh-TM4SF1 showed suppressed survival after treatment with fluorouracil. **g**. WB analysis of stemness markers (SOX2, CD44, CD133, ALDHA1) in CRC cells transfected with sh-TM4SF1. *P < 0.05

To investigate whether TM4SF1 is involved in EMT induced by TGF-β1, SW480 and LoVo cell lines were treated with recombinant human TGF-β1 protein at different concentrations (0, 10, 20 ng/mL) for 48 h. The results showed that TGF-β1 significantly promoted the migration and invasion of CRC cells **(**Fig. 3a, b and Fig. S1g, h) and increased the expression of Smad2, vimentin, N-cadherin, and MMP9 while decreasing the expression of E-cadherin **(**Fig. 3c**)**. Furthermore, we found that TM4SF1 deficiency markedly decreased the cell migration and invasion induced by TGF-β1 **(**Fig. 3a, b**),** enhanced the expression of epithelial markers (E-cadherin), and suppressed the expression of mesenchyme markers (N-cadherin, vimentin, Smad2, MMP9) at both the protein level and the mRNA level compared to that in the control group **(**Fig. 3d). These results suggested that TM4SF1 is involved in the process of EMT induced by TGF-β1 in CRC cells.

### TM4SF1 modulates stemness properties and fluorouracil resistance of CRC cells

Cancer stemness has been one of the most important potential mechanisms leading to CRC tumourigenesis and progression [21]. To elucidate whether TM4SF1 is associated with the stem cell-like properties of CRC cells, we performed a sphere formation assay and found that knockdown of TM4SF1 expression significantly decreased the sphere formation and TM4SF1 overexpression enhanced sphere formation capability of both cell lines (Fig. 3e and Fig. S2a). Further, we detected the effect of TM4SF1 on cell proliferation. The CCK-8 assay revealed that the depletion of TM4SF1 strongly diminished CRC cell growth. Conversely, TM4SF1 overexpression significantly promoted the proliferation of SW480 and LoVo cells (Fig. S2b). To further examine the function of TM4SF1 in cell resistance to chemotherapy, we treated the infected CRC cells with fluorouracil. The CCK-8 assay revealed that the viability of sh-TM4SF1-transfected cells was markedly lower than that of the control cells (Fig. 3f). In addition, we found that the IC50 value of TM4SF1 knockdown cells was 50 nmol/L, which is lower than that of the matched group. Consistently, silencing TM4SF1 significantly decreased the expression of stemness markers such as CD133, CD44, SOX2, and ALDHA1 (Fig. 3g), while TM4SF1 overexpression increased the expression of CD133 and SOX2 (Fig. S1d). These data indicated that TM4SF1 can regulate stemness and fluorouracil resistance of CRC cells.

### TM4SF1 drives EMT and cancer stemness in CRC through Wnt/β-catenin signalling

**Fig. 4:**
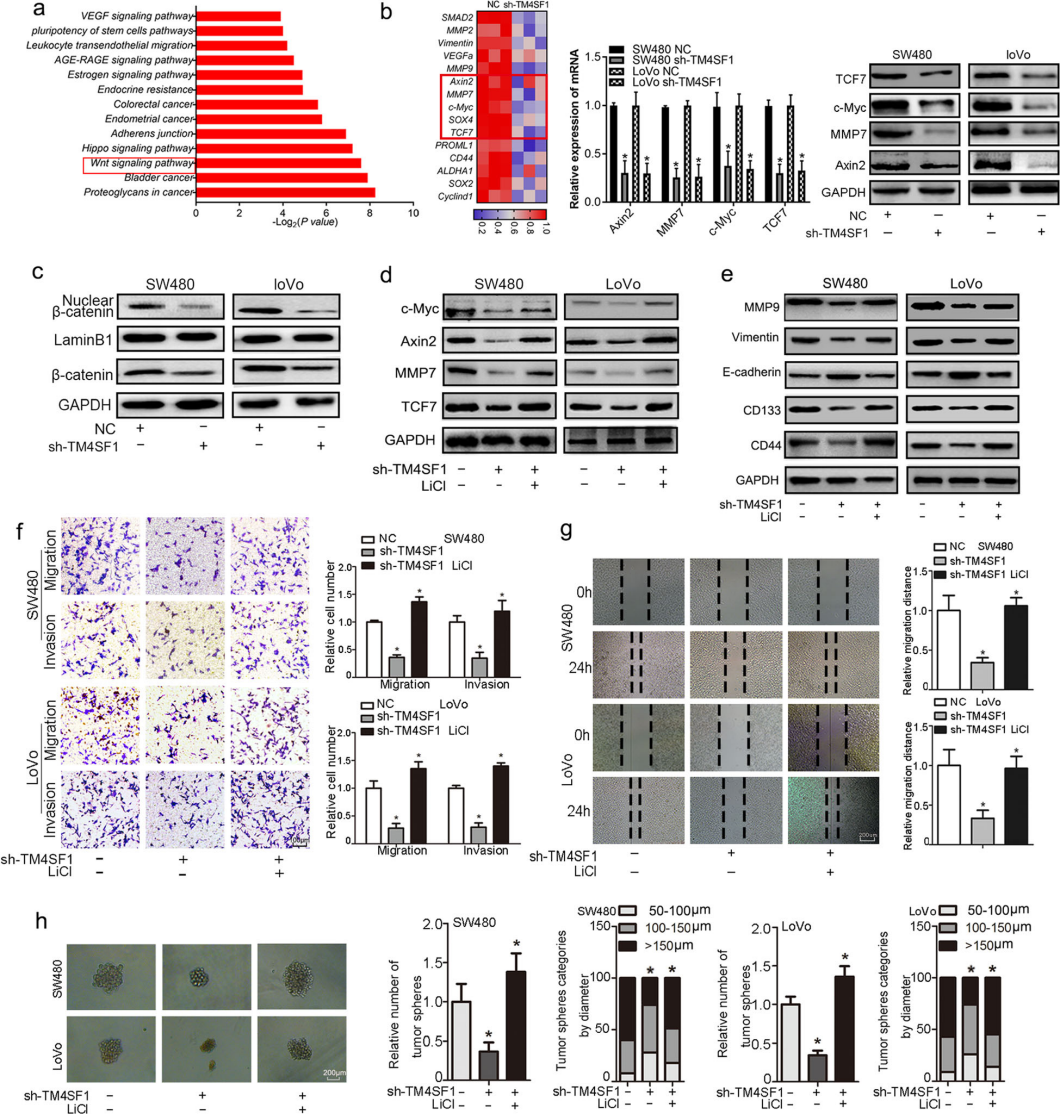
TM4SF1 drives the EMT and cancer stemness of CRC through Wnt/β-catenin signalling. **a** GSEA indicated several dysregulated Kyoto Encyclopedia of Genes and Genomes (KEGG) pathways. **b** Heat map of the indicated target genes in TM4SF1-silenced and control cells. The target genes of Wnt/β-catenin signalling were decreased in TM4SF1-silenced cells. **c** TM4SF1 knockdown reduced the levels of total β-catenin and the nuclear translocation of β-catenin. **d**, **e** LiCl restored the expression of Wnt/β-catenin target genes, EMT and stemness markers in TM4SF1-deficient cells. **f**-**g** Activation of β-catenin rescued the EMT phenotype and diminished the TM4SF1 deficiency-mediated inhibitory effect on cell migration and invasion. **h** LiCl promoted tumour sphere formation in TM4SF1-deficient cells. The tumourspheres were counted, and the percentage of tumourspheres with diameters 50–100 μm, 100–150 μm, and > 150 μm was calculated. *P < 0.05

To investigate the molecular mechanism of TM4SF1 in EMT and cancer stemness, we performed RNA-Seq to identify the differentially expressed genes (DEGs) between TM4SF1-silenced CRC cells and control cells. Gene set enrichment analysis (GSEA) indicated that the Wnt signalling pathway was one of the most impaired pathways in TM4SF1-deficient CRC cells (Fig. 4a). The depleted cells showed decreased expression of Wnt/β-catenin target genes (c-Myc, Axin2, TCF7, MMP7) (Fig. 4b). In addition, we found that TM4SF1 knockdown reduced the levels of total β-catenin and the nuclear translocation of β-catenin (Fig. 4c). Based on these observations, we evaluated whether activation of Wnt signalling was able to reverse the suppressive effects of TM4SF1 knockdown on EMT and stemness. Therefore, TM4SF1-deficient cells were treated with or without LiCl (a GSK-3β inhibitor that activates the β-catenin-mediated Wnt signalling pathway). We found that LiCl activated Wnt/β-catenin signalling and upregulated the expression of Wnt/β-catenin target genes (c-Myc, Axin2, TCF7, MMP7) in TM4SF1-deficient cells (Fig. 4d and Fig. S3a). In addition, LiCl markedly suppressed the expression of E-cadherin and enhanced the expression of MMP9, vimentin, CD133, and CD144 at both the protein and mRNA levels (Fig. 4e and Fig. S3b). Further, we found that activation of β-catenin rescued the EMT phenotype and diminished the TM4SF1 deficiency-mediated inhibitory effects on cell migration and invasion (Fig. 4f, g) and cell stemness (Fig. 4h). Then we further detected whether depletion of β-catenin or inhibition of Wnt/β-catenin pathway could reverse the promoting effect of TM4SF1 overexpression in CRC cells. The results suggested that the depletion of β-catenin and Wnt/β-catenin pathway inhibitor, XAV-393, could attenuated the regulatory effect of TM4SF1 overexpression on the expression of Wnt/β-catenin target genes (c-Myc, Axin2, TCF7, MMP7) and the EMT and stemness related markers in CRC cells (Fig. S4a, b). And sh-β-catenin or XAV-939 could markedly reduce TM4SF1-overexoression mediated migration and tumoursphere formation in SW80 and LoVo cells (Fig. S4c, d, e). These results suggested that TM4SF1 may maintain stemness and the mesenchymal phenotype in a Wnt/β-catenin signalling-dependent manner.

### TM4SF1 promotes the EMT of CRC cells via the Wnt/β-catenin/SOX2 signalling pathway

**Fig. 5:**
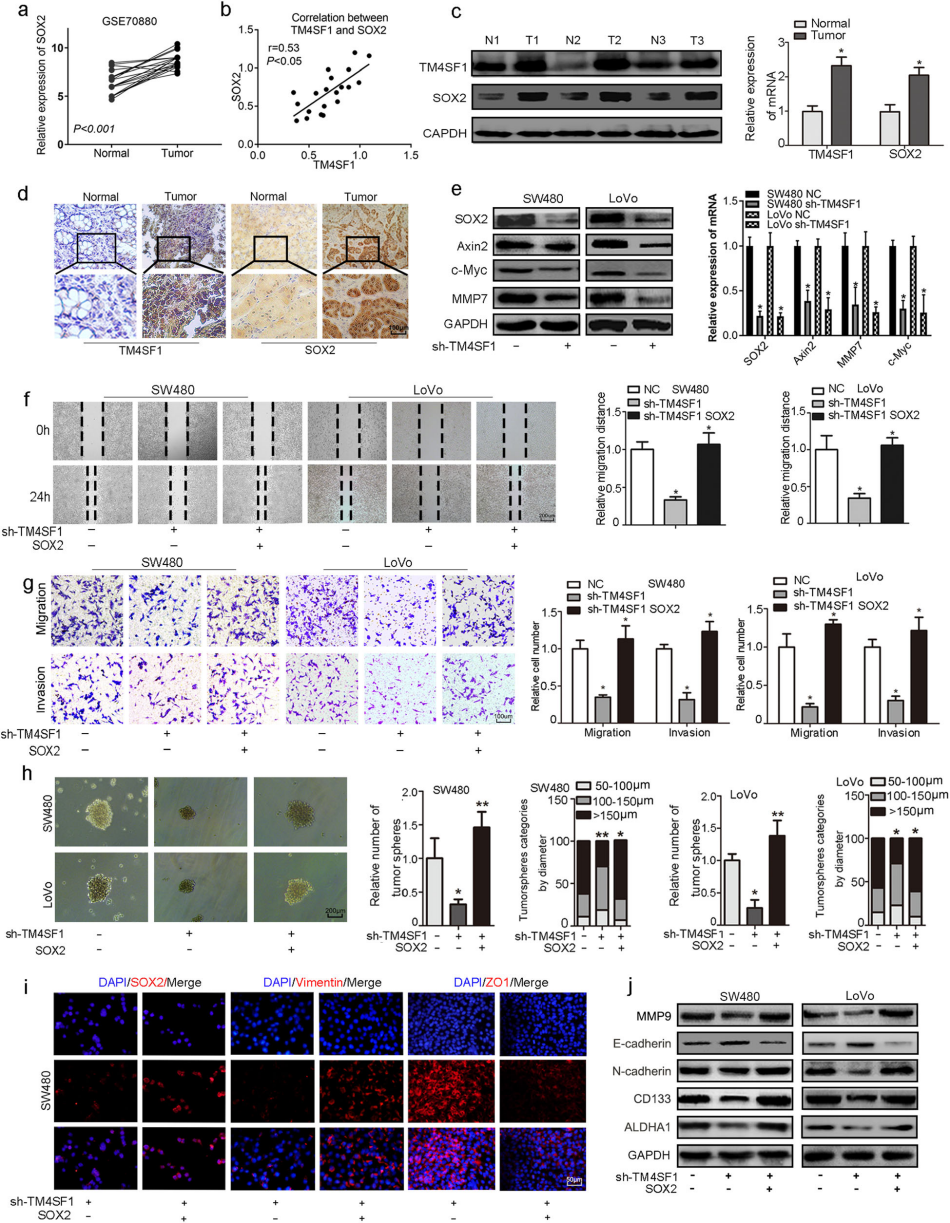
TM4SF1 promotes the EMT of CRC cells via the Wnt/β-catenin/SOX2 signalling pathway. **a** The GEO database (GSE70880) indicated that SOX2 was overexpressed in *CRC tissues.* **b** Spearman’s rank correlation analysis showed the correlation between the expression of TM4SF1 and SOX2 in CRC tissues. **c** WB and qRT-PCR analysis showed the expression of TM4SF1 and SOX2 in CRC tissues and adjacent non-tumour tissues. **d** Immunohistochemical staining for TM4SF1 and SOX2 in CRC tissues and normal tissues. **e** TM4SF1 silencing downregulated the expression of SOX2, Axin2, MMP7, and c-Myc at the protein and mRNA levels. **f** Wound healing assays showed that SOX2 overexpression rescued the inhibitory effects of migration of sh-TM4SF1-SW480/LoVo cells. **g** Transwell assays showed that SOX2 overexpression enhanced the migration and invasion potential of TM4SF1-silenced cells. **h** Tumoursphere assay of CRC cells transfected with sh-TM4SF1 cells or SOX2 cDNA. The tumoursphere number was counted, and the percentage of tumourspheres with diameters < 100 μm, 100–150 μm or > 150 μm was calculated and plotted. **i** Immunofluorescence images of SOX2, vimentin and ZO1 (red) with SOX2 overexpression in TM4SF1-deficient cells. **j** Upregulation of SOX2 expression attenuated the loss of expression of EMT and stemness markers after TM4SF1 silencing. *P < 0.05

**Fig. 6:**
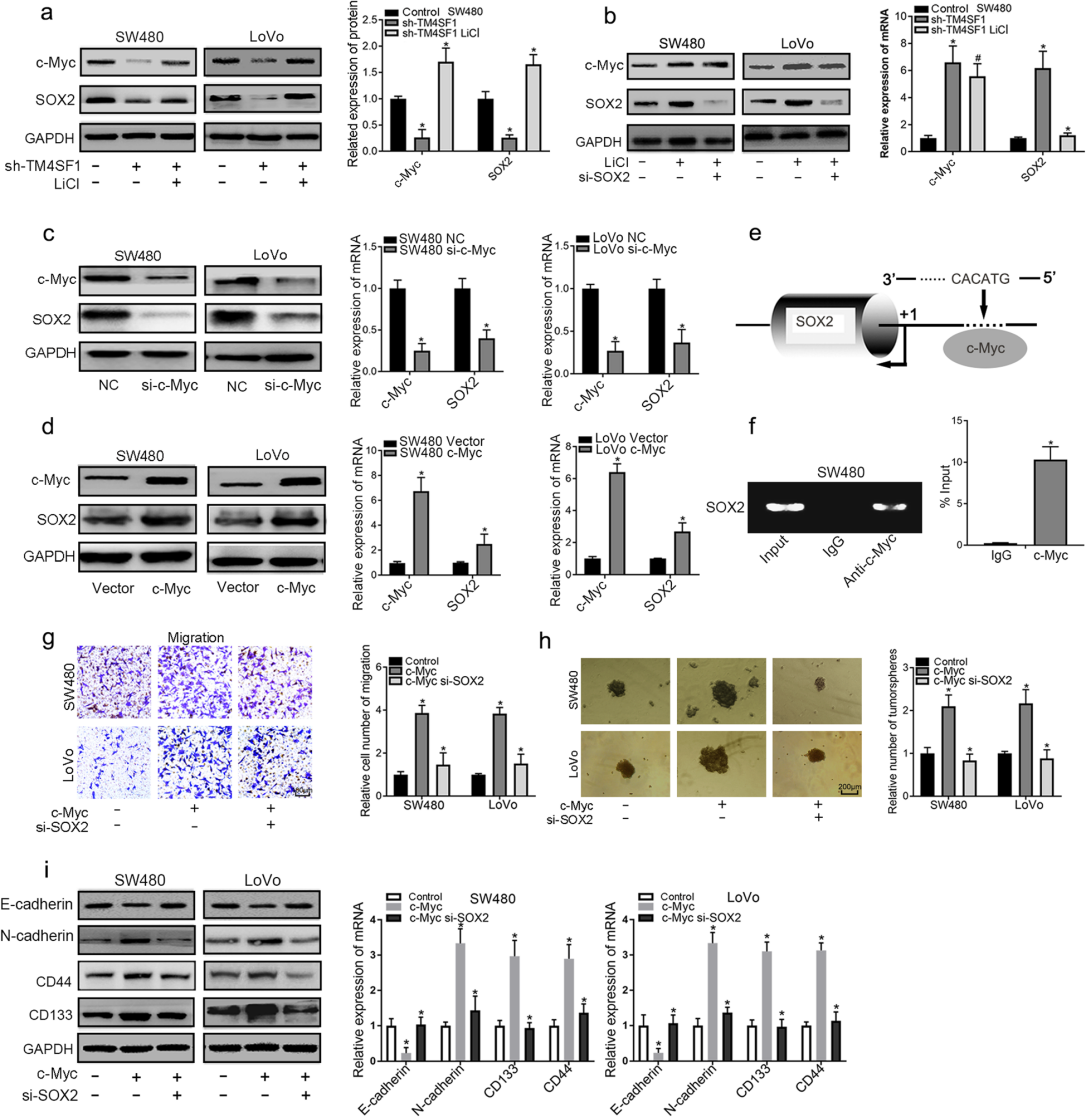
c-Myc directly binds the SOX2 promoter and regulates SOX2 expression. **a** LiCl significantly upregulated the expression of SOX2 and c-Myc in TM4SF1 knockdown CRC cells. **b** SOX2 deficiency significantly downregulated the expression of SOX2 but not the expression of c-Myc in the LiCl-treated cells. **c** c-Myc knockdown decreased the expression of SOX2. **d** c-Myc overexpression upregulated the expression of SOX2. **e** Promoter region of the SOX2 gene with a putative c-Myc binding site. **f** ChIP assays assessing the binding of c-Myc to the SOX2 promoter. Normal rabbit IgG and anti-c-Myc antibody were used to precipitate chromatin DNA fragments as indicated. **g**, **h** SOX2 knockdown suppressed migration and tumour sphere formation in c-Myc-overexpressing cells. **i** SOX2 deficiency decreased the expression of N-cadherin, CD133, and CD44 and upregulated the expression of E-cadherin in c-Myc-overexpressing cells. *P < 0.05

It has been revealed that SOX2 is overexpressed in many cancer stem progenitor cells and is involved in chemotherapy resistance and the metastasis and recurrence of cancer [28–30]. Hence, we asked whether SOX2 is necessary for the TM4SF1-induced EMT and stemness of CRC cells. We investigated the expression of SOX2 (normal vs. cancer) in the GEO database (https://www.ncbi.nlm.nih.gov/gds, GSE70880), which revealed that SOX2 was overexpressed in CRC compared to normal controls (*P* < 0.05 Fig. 5a). Spearman’s rank correlation analysis revealed that there was a strong positive correlation between the expression levels of TM4SF1 and SOX2 in CRC tissues (*r* = 0.53, *P* < 0.05, Fig. 5b). Further, we found that SOX2 was significantly overexpressed in CRC tissues compared to the paired normal tumour adjacent tissues (Fig. 5c, d). As shown in Fig. 5e, the expression of SOX2, as well as Axin2, MMP7, and c-Myc, was significantly downregulated in sh-TM4SF1 SW480/LoVo cells. Then, we constructed a stable SOX2 overexpression vector in TM4SF1 deficient cells, which showed that overexpression of SOX2 significantly potentiated the migration and invasion of SW480 and LoVo cells (Fig. 5f, g). SOX2 overexpression significantly promoted sphere formation in TM4SF1-deficient cells **(**Fig. 5h). As shown in Fig. 5i**,** compared to control cells, SW480-SOX2 cells presented decreased distribution of ZO-1 on the cell membrane and increased expression of vimentin and SOX2 in the cytoplasm. Furthermore, we found that upregulation of SOX2 rescued the TM4SF1 deficiency-mediated downregulation of N-cadherin, vimentin, MMP9, CD133, and CD44 and upregulation of the expression of E-cadherin (Fig. 5j and Fig. S5a). All these results indicated that SOX2 upregulation abolished the suppressive effect of sh-TM4SF1 on EMT and stemness. On this basis, we further investigated the effect of Wnt/β-catenin activation on the TM4SF1-mediated regulation of SOX2 expression. We treated SW480/LoVo-sh-TM4SF1 cells with LiCl as described previously. The activation of Wnt signalling restored the expression of c-Myc and SOX2, which was reduced by sh-TM4SF1 (Fig. 6a). Further, we found that SOX2 deficiency significantly downregulated the expression of SOX2 but not the expression of c-Myc in the LiCl-treated cells (Fig. 6b).

### C-Myc regulates SOX2 expression by directly binding to the SOX2 promoter in CRC cells

In the canonical Wnt/β-catenin signalling pathway, T-cell factor/Lymphoid enhancer factor (TCF/LEF) transcription factors activate the target genes by recruiting the β-catenin transcriptional coactivator and binding to the Wnt responsive DNA elements [31]. However, we did not identify a consensus TCF/LEF binding site in the promoter region of SOX2, which suggests that Wnt/β-catenin signalling may not modulate SOX2 directly and that the target genes may mediate Wnt/β-catenin/SOX2 signalling. It has been reported that c-Myc could activate SOX2 gene transcription by binding to the SOX2 promoter in lung cancer cells [32]. To further investigate the function of c-Myc in the regulation of SOX2 expression in CRC cells, SW480/LoVo cells were transiently transfected with si-c-Myc or si-Control. The results revealed that c-Myc knockdown significantly decreased the expression of SOX2 (Fig. 6c). c-Myc overexpression increased endogenous SOX2 expression at the mRNA and protein levels, suggesting that c-Myc may exert transcriptional regulation of SOX2 (Fig. 6d). Then, we identified an essential binding site for c-Myc (5′-CACATG-3′) in the SOX2 promoter (Fig. 6e), and conducted a ChIP assay with c-Myc-specific antibodies in SW480 cells to investigate whether c-Myc could bind to the sequence of the SOX2 promoter region. Cross-linked chromatin was prepared from SW480 cells, immunoprecipitation was performed using either the anti-c-Myc antibody or IgG, and the sequence containing the putative c-Myc binding site was amplified. As the figure shows (Fig. 6f), c-Myc could bind to the SOX2 promoter. In addition, we found that SOX2 knockdown restored migration and sphere formation in c-Myc-overexpressing cells (Fig. 6g, h). We found that SOX2 deficiency decreased the expression of N-cadherin, CD133, and CD44 and upregulated the expression of E-cadherin. The above results clearly demonstrated that Wnt/β-catenin/c-Myc regulates the expression of SOX2 in CRC cells (Fig. 6i).

### TM4SF1 promotes tumourigenicity and tumour metastasis in mice

**Fig. 7:**
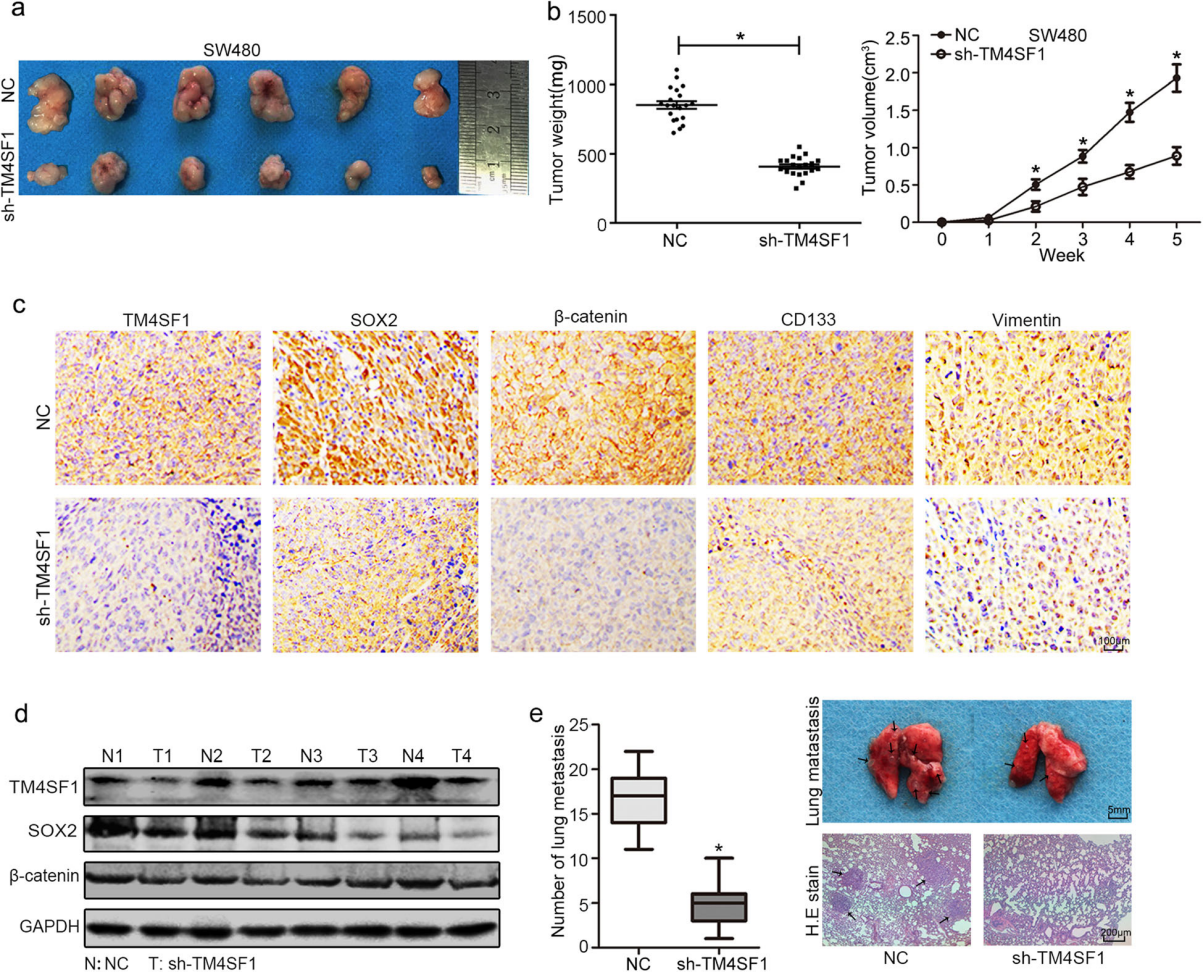
TM4SF1 promotes tumourigenesis and tumour metastasis in mice. **a**, **b** Xenograft weight (mg) and size (cm) were measured. **c** Immunohistochemistry staining of TM4SF1, SOX2, β-catenin, CD133 and vimentin in xenograft tissues. **d** WB analysis of the expression of TM4SF1, SOX2, and β-catenin expression from sh-TM4SF1/scramble xenograft tumours. **e** Abolished tumour formation was found in the lungs of SW480 cell-injected mice. The statistical data of the tumour foci number and diameter are presented, and the black arrow marked metastatic nodules. H&E stained histological images of the lungs of the two groups of mice. *P < 0.05

To validate the function of endogenous TM4SF1 in vivo, we constructed stably transfected SW480 cells, subcutaneously injected them into BALB/c-nu mice and then measured tumour growth in the xenograft mouse model weekly **(**Fig. 7a). As shown in Fig. 7b, TM4SF1 knockdown markedly suppressed tumour growth in vivo compared to that of the controls. Then we examined the expression of TM4SF1, SOX2, and β-catenin in the xenografts by IHC, WB and, qPCR; the results revealed that the xenograft derived from TM4SF1 knockdown cells showed significantly diminished expression of TM4SF1, SOX2, β-catenin, and CD133 **(**Fig. 7c, d and Fig. S6a, b). Furthermore, the effect of TM4SF1 on tumour metastasis was examined by using mouse tail-vein injection model. We determined the metastatic nodules in the lungs 6 weeks after inoculation by haematoxylin and eosin (H&E) staining, and found that compared with the control groups, both the size and number of pulmonary metastatic nodules were suppressed in the TM4SF1-knockdown groups (Fig. 7e and Fig. S6c). These results indicate that TM4SF1 is involved in tumourigenesis and tumour metastasis in vivo.

## Discussion

Although recent advances in therapy have been applied to prolong the survival rate, CRC remains one of most aggressive malignancies characterized by rapid tumour recurrence and early metastasis [33]. Recently, EMT and stemness have become widely accepted as crucial biological processes driving tumour cell invasion and metastatic dissemination from primary tumours. Growing evidence has implied that elevated expression of TM4SF1 is positively correlated with aggressive progression and occurrence in various epithelial malignant carcinomas. A wide range of studies have demonstrated that TM4SF1 exerts a promoting effect on cell proliferation and survival via JAK2/STAT3 signalling and PI3K/AKT/mTOR-related signalling pathways [9, 10]. A recent study suggested that TM4SF1 can regulate apoptosis and the cell cycle via the PARγ-SIRT1 feedback loop in bladder cancer cells [34]. Several studies have reported that TM4SF1 functions as the direct target of some miRNAs (miR-141, miR-9 miR-203) to promote the self-renewal, invasion and migration of oesophageal and breast cancer cells [13, 14]. However, the role and mechanism of TM4SF1 in CRC progression and metastasis remain largely unknown. In this study, we found that TM4SF1 expression was markedly higher in CRC tissues than in non-tumour tissues and was positively correlated with depth of invasion, T stage, lymph node metastasis, and distant metastasis in patients with CRC, which suggested that TM4SF1 may serve as a potential biomarker to predict the metastasis and prognosis of CRC.

EMT is regarded as the prerequisite step by which the initial tumour cells become motile and invasive, leading to metastasis and recurrence in many cancers [35, 36]. CSCs possess characteristics of self-renewal and multipotent differentiation, which may lead to tumourigenicity, therapeutic resistance, relapse and metastasis [37]. CSCs are able to resist chemotherapy through a series of self-defence lines. In essence, CSCs are the “roots” of aggressive tumours for which we currently have no effective treatments [35]. Accumulating studies have revealed that EMT plays an important role in the enrichment of cells with CSC properties and therapy resistance [38]. Thus, targeting biochemical composition in EMT and cancer stemness has become a frontier of cancer therapy. Jia et al. reported that TM4SF1 promoted gemcitabine resistance in pancreatic cancer by downregulating ABCB1 and ABCC1 [9]. A recent study revealed that TM4SF1 coupled with DDR1 was shown to promote cancer stem cell traits and metastatic reactivation and by PKCα-dependent JAK2/Stat3 signalling in breast cancer [37]. We found that TM4SF1 knockdown suppressed TGF-β1-induced migration, invasion and EMT. In addition, we confirmed that TM4SF1 knockdown sensitized the cells to fluorouracil and suppressed the stemness of CSCs in CRC.

There have been abundant research results on the abnormal activation of Wnt/β-catenin pathway, mostly by inactivating mutations of APC, in various human cancers, most notably colorectal carcinomas (CRCs). It is demonstrated that almost all sporadic CRCs are associated with aberrant Wnt/β-catenin signalling, whose activation could increase amount of β-catenin protein in the nucleus forms complexes with TCF/LEF to regulate target gene expression (Axin2, SOX4, TCF7, c-Myc, MMP7) [39, 40]. The dysregulation of this signalling pathway is involved in many biological processes, including proliferation, differentiation, organogenesis, and cell migration and cell autophagy. And mutations in crucial regulatory factors of the β-catenin signal is one of the most pivotal pathways contributing to EMT and self-renewal of cancer CSCs during tumour metastasis [41]. A recent study suggested that TM4SF1 could promote migration and metastasis by positively regulating the Wnt/β-catenin signalling in hepatocellular carcinoma [42]. Consistent with this research, we found that TM4SF1 deficiency significantly suppressed the stemness and EMT-associated invasion and metastasis of CRC cells by restraining the Wnt/β-catenin signalling pathway. In addition, activation of β-catenin reversed the suppressive effects of TM4SF1 silencing on stemness and EMT in vitro. Further, the depletion of β-catenin and Wnt/β-catenin pathway inhibitor, XAV-393, could significantly reversed EMT transition and tumorsphere formation induced by enhanced TM4SF1 expression. All these results validated that TM4SF1 promotes the migration, invasion and oncosphere formation of CRC cells via Wnt/β-catenin signalling pathway.

SOX2 is one of the key members of the SOX family gene and plays a critical role in the maintenance of the self-renewal and pluripotency of embryonic stem cells [43]. Studies have revealed that SOX2 is an oncogene known to be amplified and overexpressed in carcinogenesis, metastasis and recurrence in many cancer types [44, 45]. Wang et al. reported that SOX2 enhances the capability of cell migration and invasion and is associated with poor outcomes in laryngeal squamous cell carcinoma (LSCC). In addition, studies have highlighted the central roles of Wnt/β-catenin in the EMT and stemness maintenance processes, which occur via regulation of SOX2 in cancer cells [46]. A recent study revealed that TM4SF1 overexpression increases the expression of SOX2 and NANOG, sustains the manifestation of cancer stem cell traits, and drives metastatic reactivation in the lung and brain [37]. Therefore, we speculated that TM4SF1 deficiency may suppress the proficiency of EMT and sphere formation by regulating the expression of SOX2. Consistent with this speculation, we found that SOX2 overexpression reversed the downregulation of EMT or stemness hallmarks and attenuated the suppression of stemness induced by sh-TM4SF1. Further, we validated that TM4SF1 regulated the expression of SOX2 via the Wnt/β-catenin signalling pathway.

There is strong evidence showing that the activation of Wnt/β-catenin may result in the accumulation and nuclear translocation of β-catenin [47, 48]. After this occurs, T-cell factor/lymphoid enhancer factor (TCF/LEF) transcription factors bind to Wnt responsive DNA elements (WREs) and recruit a β-catenin transcriptional coactivator to activate target gene expression (Axin2, SOX4, c-Myc) [49]. However, we could not identify a consensus TCF/LEF binding site in the promoter region of SOX2, which suggests that Wnt/β-catenin target genes may mediate Wnt/β-catenin/SOX2 signalling. Recent studies have demonstrated that c-Myc is one of the key components of Wnt signalling target genes, and overexpression of c-Myc has been linked to both EMT and CSC properties in the development of CRC. Additionally, high expression of c-Myc could maintain cancer stemness by increasing the transcriptional activity of SOX2 in lung cancer cells [50]. Therefore, we speculate that c-Myc could regulate SOX2 expression. Karen I. Zeller first identified the global genomic view of Myc binding sites, which provided a substantial understanding of transcriptional circuitries and cis regulatory modules of Myc-induced tumourigenesis [51]. This study characterized the properties of Myc–DNA interactions by using the motif discovery algorithm Weeder and detected two novel consensus motifs for Myc, 5′-CACGTG-3′ (the canonical motif) and 5′-CACATG-3′ (the noncanonical motif). Interestingly, we identified the noncanonical E box, 5′-CACATG-3′, in the sequence of the SOX2 promoter. Therefore, we performed a ChIP assay and found that c-Myc could bind to the promoter of SOX2, suggesting that c-Myc regulates SOX2 expression by increasing recruitment to the SOX2 promoter. In our study, we found that TM4SF1 modulates the Wnt/β-catenin -mediated regulation of Sox2 expression via c-Myc in CRC.

## Conclusion

In conclusion, we identified a promising EMT and CSC-targeted therapeutic gene: TM4SF1. High TM4SF1 expression is positively correlated with T classification, lymph node metastasis and predicts an unfavourable prognosis for CRC patients. Mechanistically, this study revealed that TM4SF1 could facilitate cell migration, invasion and maintains stemness via the Wnt/β-catenin/c-Myc/SOX2 axis in CRC. This novel signaling axis that functions in CRC tumorigenesis provides a better understanding regarding the role of Wnt/β-catenin signaling pathways and the crosstalk between SOX2 and β-catenin in the progression of CRC. Overall, these data indicated that TM4SF1 may be a potential prognostic marker for predicting metastasis and a target for individualized drug therapy, which may prevent tumour metastasis and improve the prognosis of cancer patients.

## Supplementary information

**Additional file 1: Fig. S1.** (a) TM4SF1 cDNA transfection significantly increased the expression of TM4SF1 in SW480 and LoVo cells. (b, c) TM4SF1 overexpression increased the migration and invasion potential of CRC cells. (d) TM4SF1 maintained EMT and stemness with increased expression of β-catenin, vimentin, and CD133, while E-cadherin was decreased. *P < 0.05. (e). Immunofluorescence staining showed that TM4SF1 overexpression resulted in decreased expression of ZO-1 and increased expression of vimentin. (f) TM4SF1-overexpressing cells exhibited a more mesenchymal phenotype than control cells, as observed under a phase contrast microscope. (g) Wound healing assays showed that TGF-β1 significantly enhanced the migration potential of LoVo cells. TM4SF1 silencing decreased the migration of TGF-β1-treated LoVo cells. (h) Transwell assays showed that TGF-β1 significantly enhanced the migration and invasion potential, and TM4SF1 silencing suppressed the migration and invasion of TGF-β1-treated LoVo cells. *P < 0.05.

**Additional file 2: Fig. S2.** (a) Sphere formation assay showed that TM4SF1 overexpression enhanced sphere formation in SW480 and LoVo cells. (b) The CCK-8 assay revealed that the depletion of TM4SF1 strongly diminished CRC cell growth. Conversely, TM4SF1 overexpression significantly promoted the proliferation of CRC cells, *P < 0.05.

**Additional file 3: Fig. S3.** (a) Activation of β-catenin with LiCl upregulated the expression of Wnt/β-catenin target genes (c-Myc, Axin2, TCF7, MMP7) in TM4SF1-deficient cells. (b) qRT-PCR analysis of the expression of EMT and stemness markers after LiCl treatment.

**Additional file 4: Fig. S4.** (a, b) WB and qRT-PCR analysis revealed that silencing β-catenin by shRNA or inhibition of β-catenin with XAV-939 (a β-catenin inhibitor) treatment could attenuated the regulatory effect of TM4SF1 overexpression on the expression of Wnt/β-catenin target genes (c-Myc, Axin2, TCF7, MMP7) and the EMT and stemness related markers in CRC cells. (c, d) Wound healing and Transwell assay showed that sh-β-catenin or XAV-939 could markedly reduce TM4SF1-overexoression mediated migration in SW80 and LoVo cells. (e) β-catenin silencing or inhibition partly counteracted the effects of TM4SF1-overexpression on sphere formation in CRC cells. Data are represented as mean ± SD of three independent, * *p* < 0.05.

**Additional file 5: Fig. S5**. (a) qRT-PCR analysis showed that SOX2 overexpression attenuated the loss of EMT and stemness markers with increased expression of MMP9, N-cadherin, CD133, CD44 and decreased expression of E-cadherin. *P < 0.05.

**Additional file 6: Fig. S6**. (a, b) WB and qRT-PCR analysis of the expression of TM4SF1, SOX2, and β-catenin expression from sh-TM4SF1/sh-Control xenograft tumours. (c) Abolished tumour formation was found in the lungs of SW480 cell-injected mice. The statistical data of the tumour diameter are presented, *P < 0.05.

**Additional file 7: Table S1**. Source of Antibodies and Reagents. **Table S2**. Primer sets used for RT-PCR.

## Data Availability

The datasets analyzed during the current study are not publicly available but are available from the corresponding author on reasonable request.

## Abbreviations

TGF-β1: Transforming growth factor beta 1
DAPI: 4′, 6- diamidi-no-2-phenylindole
qRT-PCR: Quantitative reverse transcription polymerase chain reaction
CSC: Cancer stem cell
CRC: Colorectal cancer
TM4SF1: Transmembrane 4 L six family member 1
EMT: Epithelial to mesenchymal transition
ECM: Extracellular matrix
IHC: Immunohistochemistry
CHIP: Chromatin Immunoprecipitation
MET: Mesenchymal-epithelial transition
FBS: Fetal bovine serum
TCGA: The Cancer Genome Atlas database
SOX2: SRY-Box Transcription Factor 2
WREs: Wnt responsive DNA elements
TMED: TM4FS1-enriched domains
mRNA: Messenger RNA
shRNA: Short hairpin RNA
