# Supplementary figures and images for: TM4SF1 promotes EMT and cancer stemness via the Wnt/β-catenin/SOX2 pathway in colorectal cancer
Source: J Exp Clin Cancer Res. 2020 Nov 5;39:232. doi: 10.1186/s13046-020-01690-z (PMC7643364; doi:10.1186/s13046-020-01690-z)

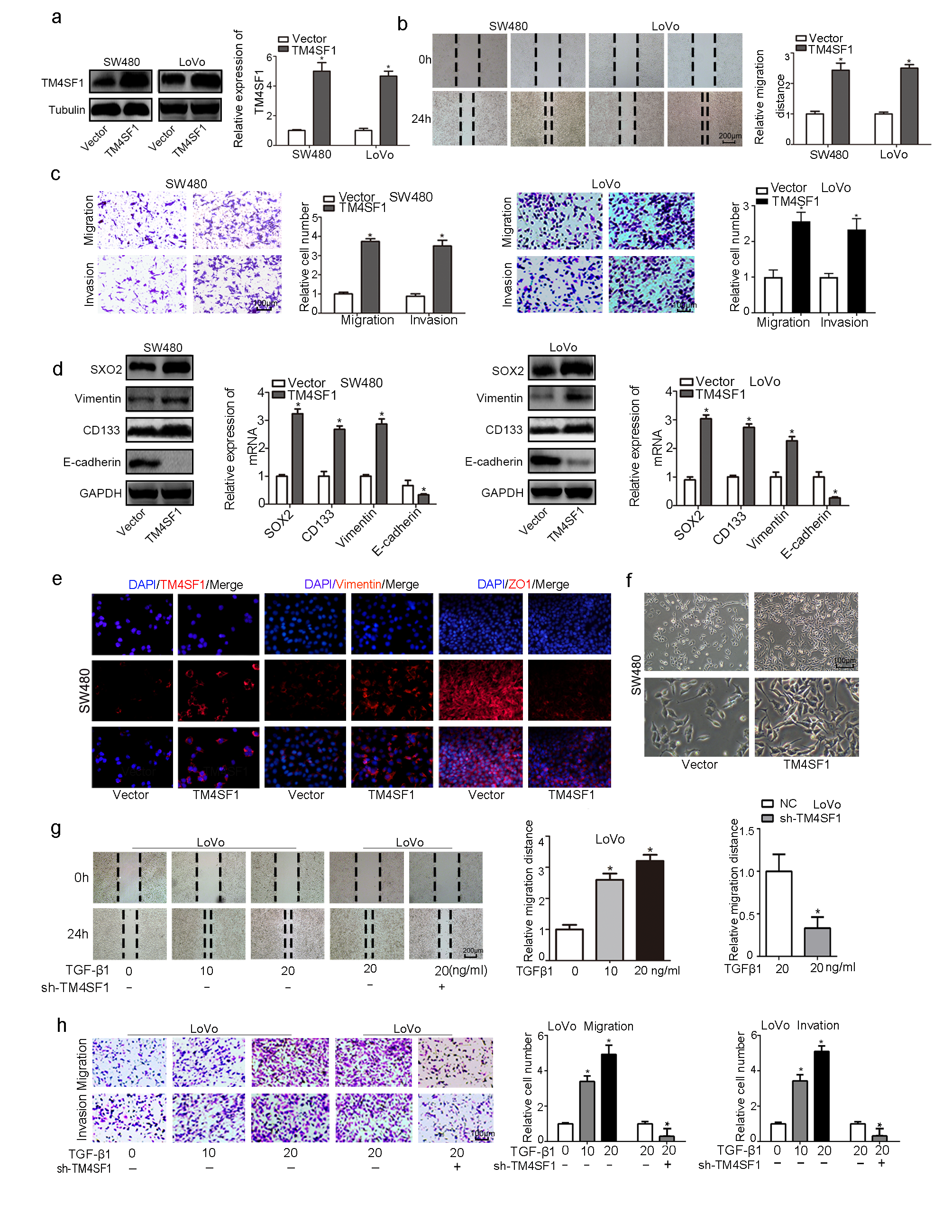

Supplement: Supplementary file 1 — Additional file 1: Fig. S1. (a) TM4SF1 cDNA transfection significantly increased the expression of TM4SF1 in SW480 and LoVo cells. (b, c) TM4SF1 overexpression increased the migration and invasion potential of CRC cells. (d) TM4SF1 maintained EMT and stemness with increased expression of β-catenin, vimentin, and CD133, while E-cadherin was decreased. *P < 0.05. (e). Immunofluorescence staining showed that TM4SF1 overexpression resulted in decreased expression of ZO-1 and increased expression of vimentin. (f) TM4SF1-overexpressing cells exhibited a more mesenchymal phenotype than control cells, as observed under a phase contrast microscope. (g) Wound healing assays showed that TGF-β1 significantly enhanced the migration potential of LoVo cells. TM4SF1 silencing decreased the migration of TGF-β1-treated LoVo cells. (h) Transwell assays showed that TGF-β1 significantly enhanced the migration and invasion potential, and TM4SF1 silencing suppressed the migration and invasion of TGF-β1-treated LoVo cells. *P < 0.05. [file 13046_2020_1690_MOESM1_ESM.tif]

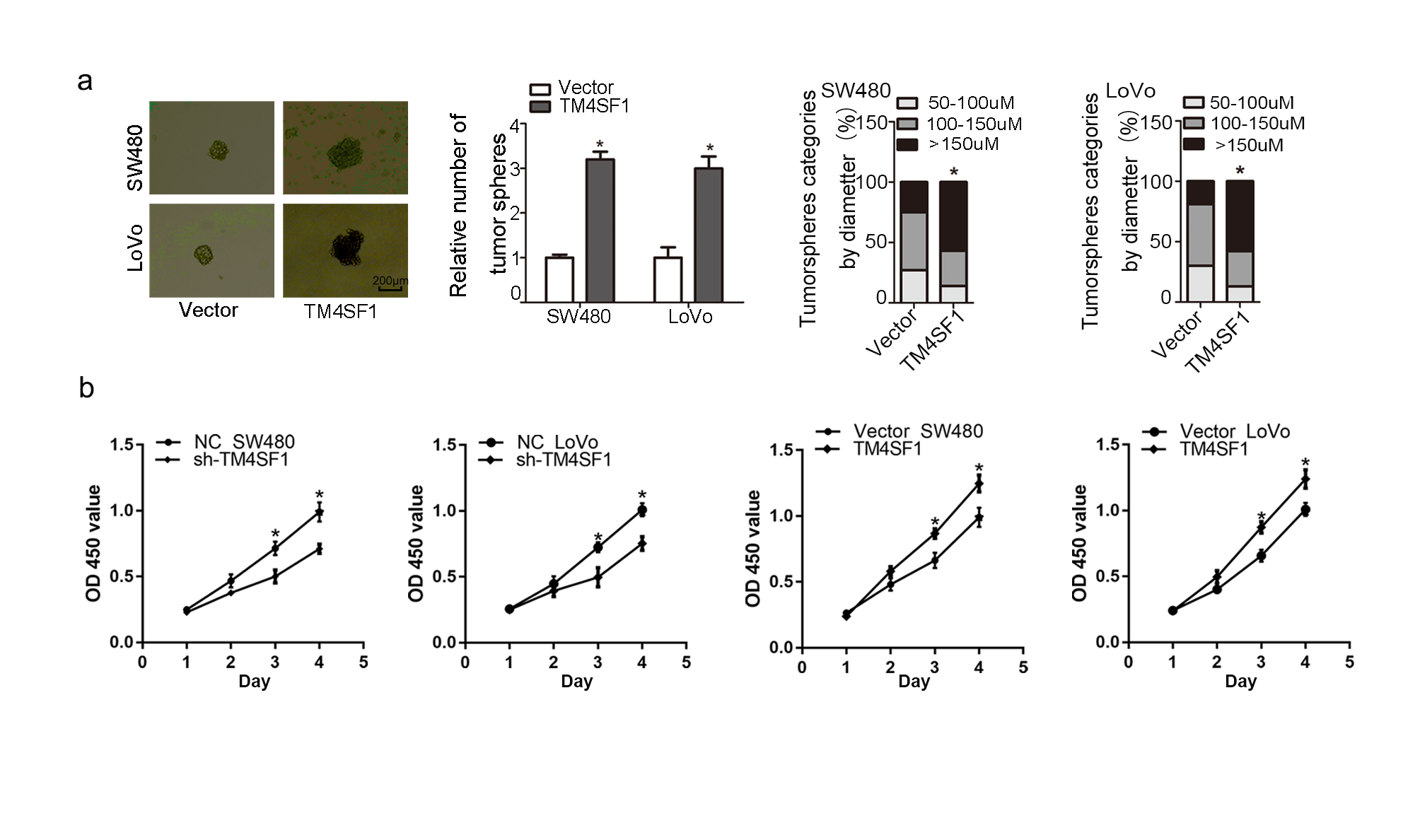

Supplement: Supplementary file 2 — Additional file 2: Fig. S2. (a) Sphere formation assay showed that TM4SF1 overexpression enhanced sphere formation in SW480 and LoVo cells. (b) The CCK-8 assay revealed that the depletion of TM4SF1 strongly diminished CRC cell growth. Conversely, TM4SF1 overexpression significantly promoted the proliferation of CRC cells, *P < 0.05. [file 13046_2020_1690_MOESM2_ESM.tif]

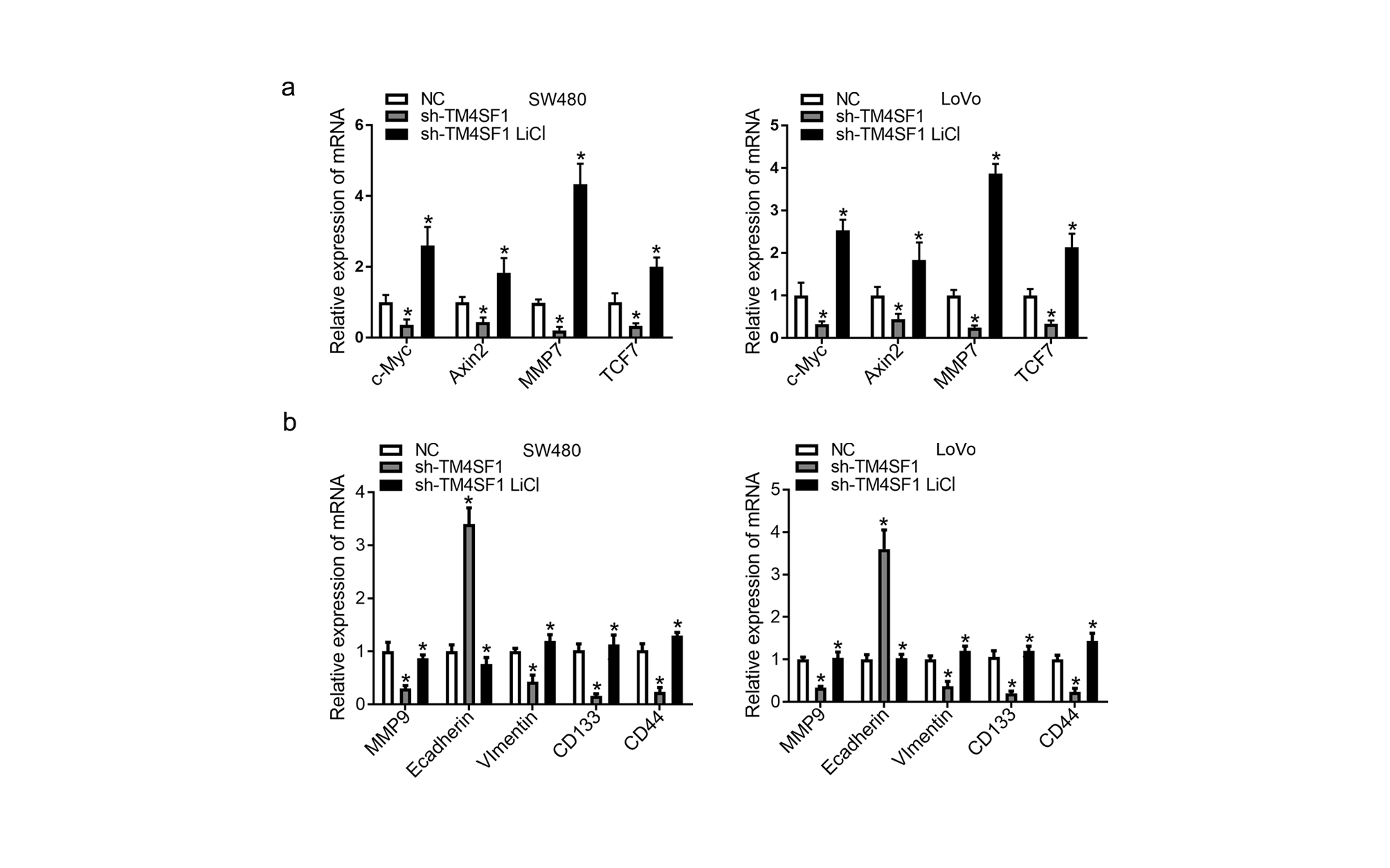

Supplement: Supplementary file 3 — Additional file 3: Fig. S3. (a) Activation of β-catenin with LiCl upregulated the expression of Wnt/β-catenin target genes (c-Myc, Axin2, TCF7, MMP7) in TM4SF1-deficient cells. (b) qRT-PCR analysis of the expression of EMT and stemness markers after LiCl treatment. [file 13046_2020_1690_MOESM3_ESM.tif]

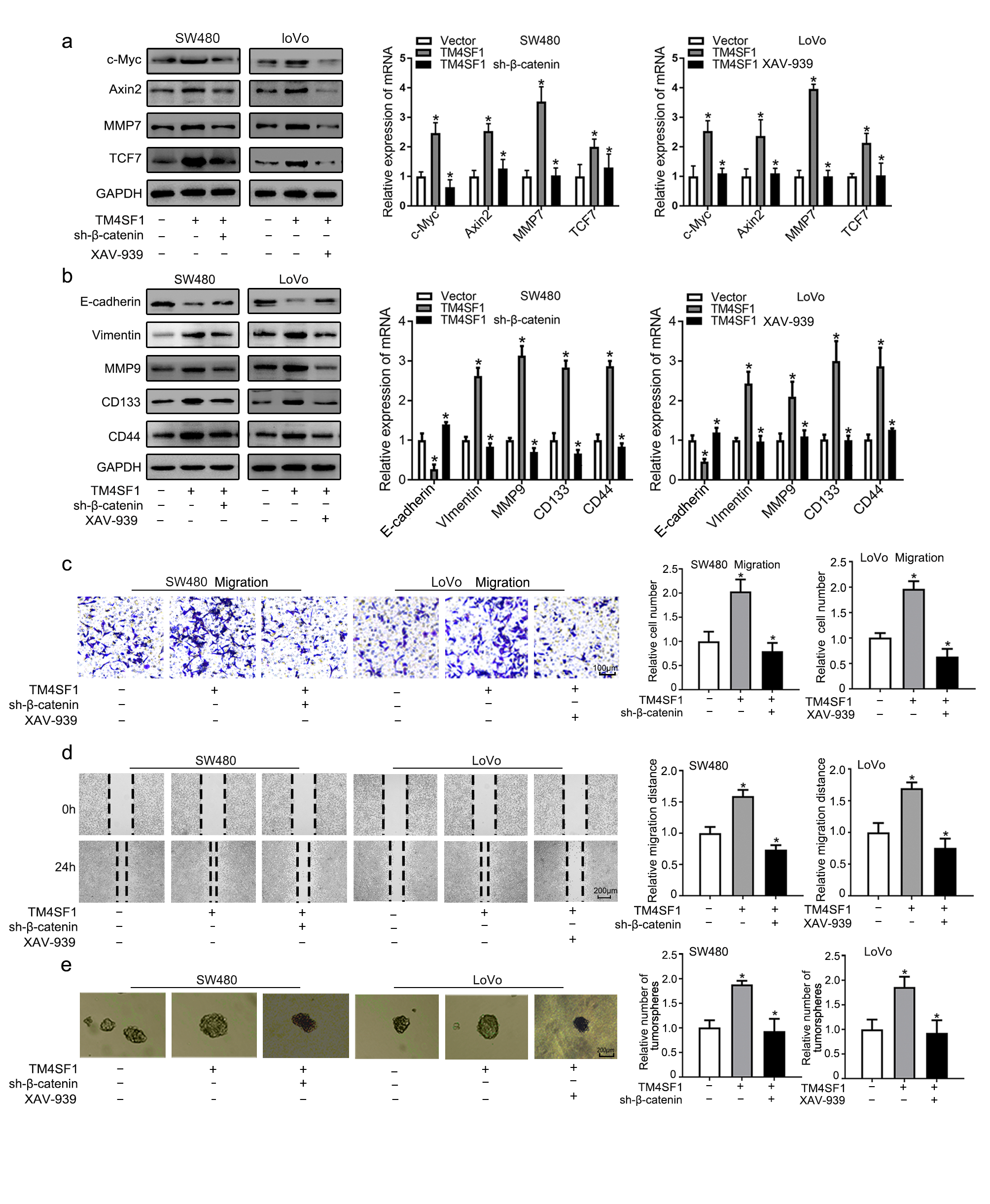

Supplement: Supplementary file 4 — Additional file 4: Fig. S4. (a, b) WB and qRT-PCR analysis revealed that silencing β-catenin by shRNA or inhibition of β-catenin with XAV-939 (a β-catenin inhibitor) treatment could attenuated the regulatory effect of TM4SF1 overexpression on the expression of Wnt/β-catenin target genes (c-Myc, Axin2, TCF7, MMP7) and the EMT and stemness related markers in CRC cells. (c, d) Wound healing and Transwell assay showed that sh-β-catenin or XAV-939 could markedly reduce TM4SF1-overexoression mediated migration in SW80 and LoVo cells. (e) β-catenin silencing or inhibition partly counteracted the effects of TM4SF1-overexpression on sphere formation in CRC cells. Data are represented as mean ± SD of three independent, * p < 0.05. [file 13046_2020_1690_MOESM4_ESM.tif]

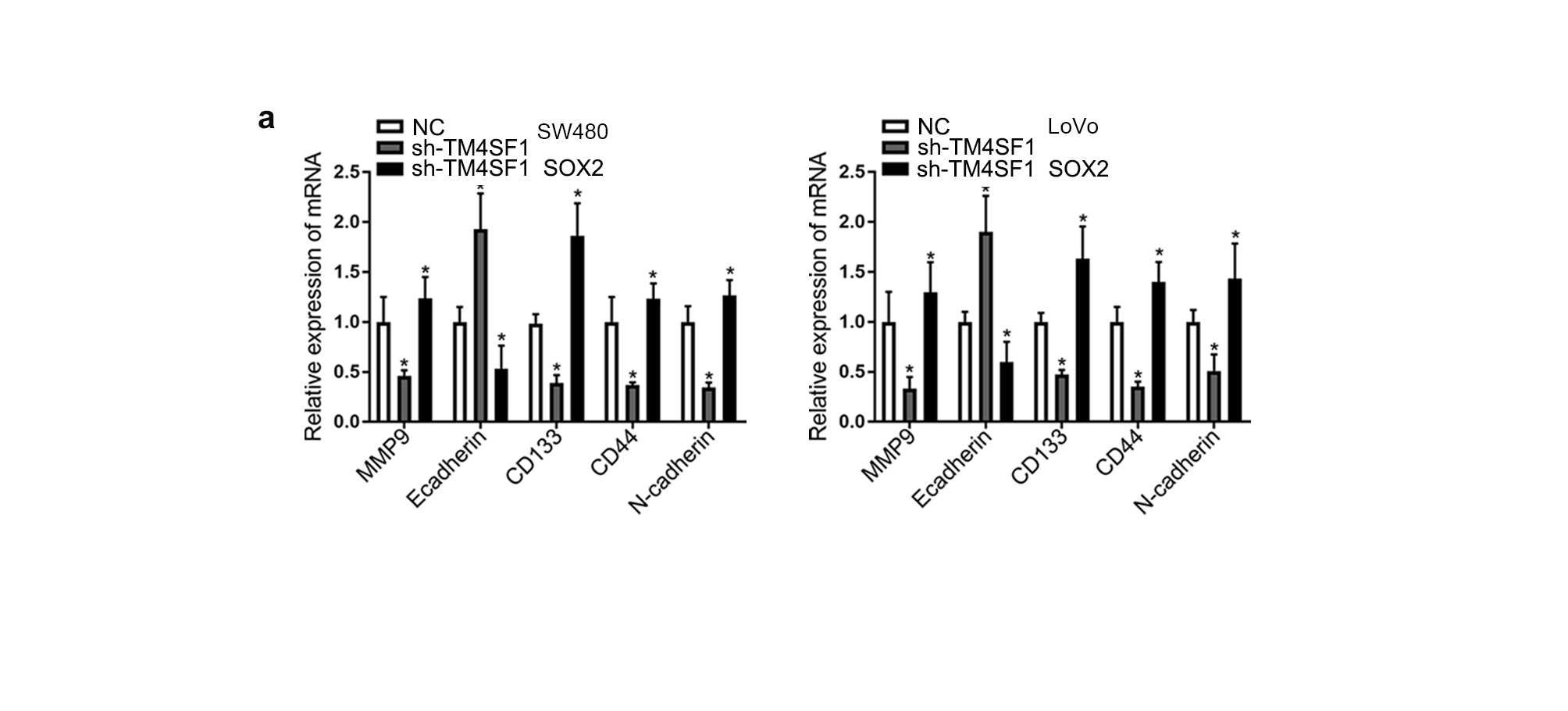

Supplement: Supplementary file 5 — Additional file 5: Fig. S5. (a) qRT-PCR analysis showed that SOX2 overexpression attenuated the loss of EMT and stemness markers with increased expression of MMP9, N-cadherin, CD133, CD44 and decreased expression of E-cadherin. *P < 0.05. [file 13046_2020_1690_MOESM5_ESM.tif]

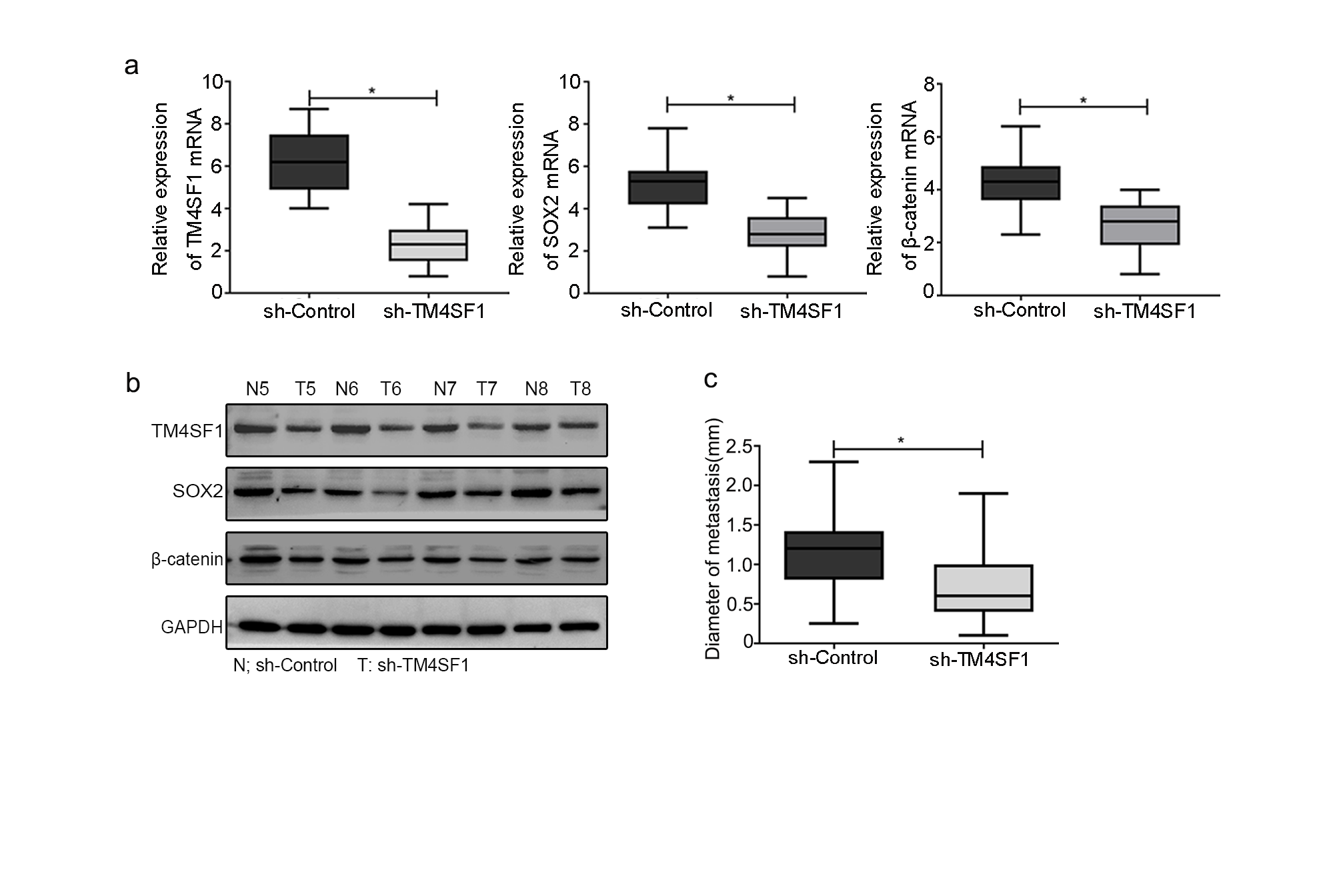

Supplement: Supplementary file 6 — Additional file 6: Fig. S6. (a, b) WB and qRT-PCR analysis of the expression of TM4SF1, SOX2, and β-catenin expression from sh-TM4SF1/sh-Control xenograft tumours. (c) Abolished tumour formation was found in the lungs of SW480 cell-injected mice. The statistical data of the tumour diameter are presented, *P < 0.05. [file 13046_2020_1690_MOESM6_ESM.tif]
