# Supplementary material for: TM4SF1 promotes EMT and cancer stemness via the Wnt/β-catenin/SOX2 pathway in colorectal cancer
Source: J Exp Clin Cancer Res. 2020 Nov 5;39:232. doi: 10.1186/s13046-020-01690-z (PMC7643364; doi:10.1186/s13046-020-01690-z)
Supplement: Supplementary file 7 — Additional file 7: Table S1. Source of Antibodies and Reagents. Table S2. Primer sets used for RT-PCR. [file 13046_2020_1690_MOESM7_ESM.docx]

**Table S1: Source of** **Antibodies and Reagents.**

| **Antibodies or reagents** | Source | Catalog numbers |
| --- | --- | --- |
| GAPDH | Abcam, USA | ab9485 |
| Tubulin | Abcam, USA | ab6046 |
| TM4SF1 | Abcam, USA | ab113504 |
| E-cadherin | Abcam, USA | ab197751 |
| CD44 | Sigma, USA | MABF581 |
| Vimentin | Abcam, USA | ab92547 |
| MMP9 | Proteintech Group, China | 10375-2-AP |
| Bcl-2 | Abcam, USA | ab692 |
| Smad2 | Sigma-Aldrich, USA | SAB4200354 |
| SOX2 | Abcam, USA | ab79351 |
| N-cadherin | Abcam, USA | ab18203 |
| CD133 | Abcam, USA | ab216323 |
| Par3 | Proteintech Group, China | 11085-1-AP |
| ZO1 | Proteintech Group, China | 21773-1-AP |
| c-Myc | Sigma-Aldrich, USA | M4439 |
| ALDHA1 | Abcam, USA | ab9883 |
| MMP7 | Proteintech Group, China | 10374-2-AP |
| TCF7 | Proteintech Group, China | 13838-1-AP |
| β-catenin | Proteintech Group, China | 51067-2-AP |
| Anti-IgG H&L (HRP) | Abcam, USA | ab6734 |
| TM4SF1 shRNA | Shanghai Gene Chem Co, Ltd, China | NM_011334 |
| SOX2-cDNA plasmids | Shanghai Gene Chem Co, Ltd, China | NM_011443 |
| TM4SF1-cDNA plasmids | Shanghai Gene Chem Co, Ltd, China | NM_014220 |
| Matrigel | Sigma-Aldrich， | E1270 |
| Primers | Sangon Shanghai, China | None |
| Cell Counting Kit (CCK-8） | Sevenbio | SC119-01 |
| DMEM/F-12 | Gibco, Invitrogen, USA | A4192001 |
| EGF | Gibco, Invitrogen, USA | PHG0311 |
| β-FGF | Gibco, Invitrogen, USA | KHG0021 |
| DAPI Stain Solution | Yeasen Biotech Co., Ltd. China | 40728ES03 |
| EZ-ChIP™ kit | Millipore，USA | 17-408 |
| XAV939 | SELLECK | S1180 |

**Table S2: Primer sets used for RT-PCR**

| GAPDH | Forward | GGGGAGCCAAAAGGGTCATCATCT |
| --- | --- | --- |
|  | Reverse | GACGCCTGCTTCACCACCTTCTTG |
| TM4SF1 | Forward | GTGGAGGAAATTGTGGCTGT |
|  | Reverse | CGTTCATGGTGATCCAACTG |
| E-cadherin | Forward | GCCCTGCCAATCCCGATGAAA |
|  | Reverse | GGGGTCAGTATCAGCCGCT |
| Vimentin | Forward | GCTTCAGAGAGAGGAAGCCGAAAA |
|  | Reverse | CCGTGAGGTCAGGCTTGGAAA |
| MMP9 | Forward | CAGAGATGCGTGGAGAGT |
|  | Reverse | TCTTCCGAGTAGTTTTGG |
| Bcl-2 | Forward | TGTGTGGAGAGCGTCAAC |
|  | Reverse | ACAGCCAGGAGAAATCAA |
| CyclinD1 | Forward | GAACACGGCTCACGCTTAC |
|  | Reverse | CCCAGACCCTCAGACTTGC |
| SOX2 | Forward | TGGACAGTTACGCGCACAT |
|  | Reverse | CGAGTAGGACATGCTGTAGGT |
| N-cadherin | Forward | AGCCAACCTTAACTGAGGAGT |
|  | Reverse | GGCAAGTTGATTGGAGGGATG |
| CD133 | Forward | AGTCGGAAACTGGCAGATAGC |
|  | Reverse | GGTAGTGTTGTACTGGGCCAAT |
| Par3 | Forward | CAACTGGCCGCATTTAAGCC |
|  | Reverse | CAGTGGAGTGCCTAGTTTTAGAG |
| ZO1 | Forward | ACCAGTAAGTCGTCCTGATCC |
|  | Reverse | TCGGCCAAATCTTCTCACTCC |
